# Bibliometric analysis of research on retinoic acid in the field of kidney disorders

**DOI:** 10.3389/fphar.2024.1435889

**Published:** 2024-08-15

**Authors:** Yu Liu, Dongxuan Sun, Youqun Huang, Yuli Shen, Tong Chen, Wenya Chen, Liangjun Zhu, Fang Wang, Guoai Hong, Yuechan Luo, Siyu Long, Hequn Zou

**Affiliations:** ^1^ Department of Nephrology, South China Hospital of Shenzhen University, Shenzhen, China; ^2^ Guangdong Key Laboratory for Biomedical Measurements and Ultrasound Imaging, National-Regional Key Technology Engineering Laboratory for Medical Ultrasound, School of Biomedical Engineering, Shenzhen University Medical School, Shenzhen, China; ^3^ Department of Nephrology, Hospital of Chengdu University of Traditional Chinese Medicine, Chengdu, China; ^4^ Nephrology Depariment of The Second Affiliated Hospital, School of Medicine, The Chinese University of Hong Kong, Shenzhen, China; ^5^ Department of Oncology, The Third Affiliated Hospital of Guizhou Medical University, Duyun, China; ^6^ Medical School, The Chinese University of Hong Kong, Shenzhen, China

**Keywords:** bibliometrics, retinoic acid, kidney disease, zebrafish, organ development

## Abstract

Retinoic acid is an active metabolite with significant physiological functions in human development, immunity, vision, and skin health. In recent years, research on retinoic acid in the field of kidney disorders has been increasing gradually. Yet, there is a lack of systematic bibliometric analysis of retinoic acid research in the kidney domain. This study included 1,368 articles published between 1998 and 2023 on treating kidney diseases with retinoic acid. Using the bibliometric analysis software VOSviewer and CiteSpace, we analyzed data on publication trends, contributing countries and institutions, journals and cocited journals, authors and cocited authors, cocited references, research hotspots, and frontiers. On the basis of the results of the bibliometric analysis, we identified the research efforts and their developmental trends, providing the groundwork for future research on retinoic acid.

## 1 Introduction

Retinoic acid (RA) is derived from retinol (vitamin A), a metabolite. RA exists in various isomeric forms, including all-trans RA, 9-cis RA, and 13-cis RA; however, all-trans RA is the primary ligand during development (Fields et al., 2007). Mechanisms of retinoic acid signaling and its roles in organ and limb development. Nature Rev. Mol. Cell Biol. 16, 110-123). Early studies on the induction of vitamin A deficiency in mammals or birds revealed the critical role of retinol (and potentially RA) in the development of various organs, such as the hindbrain, spinal cord, forelimb buds, bones, heart, eyes, pancreas, lungs, and urogenital tract (Clagett-Dame and DeLuca, 2002). Research has shown that RA is crucial for embryonic development in chordates (Marlétaz et al., 2006). However, some nonchordate animals may possess nuclear receptors similar to RA receptors (RARs) (Handberg-Thorsager et al., 2018), there is no conclusive evidence that RA is essential for the development of nonchordate animals. In addition to vitamin A deficiency, genetic research is vital for identifying processes dependent on RA, as the distinctions between genetic dysfunction and pharmacological manipulation of RA signaling make it challenging to identify specific developmental processes requiring RA (Rhinn and Dollé, 2012; Cunningham and Duester, 2015).

Retinoic acid participates in cellular proliferation, differentiation, and apoptosis (Fields et al., 2007). ATRA regulates gene expression transcriptionally by binding to the retinoic acid receptor (RAR) and the retinoic acid X receptor (RXR), thereby influencing cell growth and differentiation. RAR and RXR in humans are classified into three distinct subtypes, namely, alpha, beta, and gamma, each with multiple subtypes that vary in function and tissue distribution, thereby activating different genes. Pharmaceutical research can inadvertently affect the expression of RA-dependent genes, given that the concentrations of exogenous RA or RAR antagonists are typically approximately 1000 times higher than endogenous RA levels (Horton and Maden, 1995). In 1995, the US Food and Drug Administration (FDA) endorsed the use of oral pharmacological ATRA concentrations in patients with APL. This endorsement followed Breitman and colleagues’ 1980 study, which highlighted the potential of ATRA to induce *in vitro* differentiation of APL-derived cells (Breitman et al., 1980). In 1987, Huang and colleagues conducted clinical trials. They reported that ATRA achieved complete remission in APL patients (Huang et al., 1987). To date, the treatment of APL with oral ATRA concentrations remains the standard of care (Sanz et al., 2019). Molecular studies have revealed that most APL cases are characterized by chromosomal translocations, resulting in chimeric fusion proteins, primarily the PML–RARα fusion protein. In these cells, the PML–RARα protein is abundantly present and nonfunctional compared with the wild-type RARα protein. Functioning as an inhibitor requires pharmacological ATRA levels to overcome this inhibition and facilitate the expression of ATRA target genes (Jing, 2004). To date, APL remains the only cancer type in which a 95% complete remission rate can be achieved through the combination of chemotherapy and natural retinoids (ATRA). Skin T-cell lymphoma also shows some positive responses, but the synthetic RXR-selective retinol saxitroban has been approved by the FDA since 2009 for the systemic treatment of skin T-cell lymphoma (Sokołowska-Wojdyło et al., 2013).

Kidney disease is a global issue, with approximately three million people diagnosed and up to one million people undiagnosed in the United Kingdom alone (Outtandy et al., 2019). Factors such as obesity, diabetes, and hypertension have been shown to contribute to renal dysfunction, yet further research is necessary to understand the underlying pathophysiology. Kidney diseases can be classified as acute kidney injury (AKI) or chronic kidney disease (CKD), both of which are linked to high morbidity and mortality. Acute kidney injury is characterized by a swift and reversible decline in renal function, which is linked to the acceleration of chronic kidney disease (Siew and Davenport, 2015). The capacity to diagnose acute kidney injury has markedly improved. Recent consensus diagnostic criteria include a serum creatinine increase of ≥0.3 mg/dL (≥26.5 μmol/L) within 48 h, an elevation in serum creatinine to more than 1.5 times the baseline, or a urine output of <0.5 mL/kg/h sustained over 6 h (Khwaja, 2012). Various risk factors, including drugs/toxins, sepsis, and ischemia‒reperfusion (IR), often cause acute kidney injury, leading to a reduction in the glomerular filtration rate (GFR) and acute tubular cell death (Sanz et al., 2013). CKD represents a significant global medical issue, with the incidence rapidly increasing due to the increase in hypertension and diabetes (Tomino, 2014). CKD is commonly diagnosed by the presence of proteinuria or an estimated glomerular filtration rate with a serum creatinine level less than 60 mL/min/1.73 m^2^ (Andrassy, 2013). Treatment options for these diseases are limited. Steroids and immunosuppressive drugs serve as first-line treatments for glomerular diseases. However, drug resistance is frequently observed, and the side effects of these treatments vary. Treatment of glomerular diseases with angiotensin-converting enzyme inhibitors or angiotensin receptor blockers can reduce proteinuria and slow the progression of kidney disease. However, they afford only partial protection. Therefore, identifying new therapeutic targets or strategies is critical. During kidney development, retinoic acid influences the growth of renal tubules and the quantity of nephrons (Merlet-Bénichou et al., 1999). In recent years, research on the use of retinoic acid in nephrology has gradually increased. Some studies have shown that retinoic acid is effective against kidney diseases because it can inhibit kidney damage and fibrosis (DiKun et al., 2024), repair mesangial cells (Suzuki et al., 2003), and reduce renal inflammatory responses (Chen et al., 2021). In a rat model induced by puromycin aminonucleoside for nephropathy, retinoids prevent proteinuria by safeguarding podocytes from harm (Moreno-Manzano et al., 2003). Treatment with isotretinoin notably mitigates glomerular damage in rats with chronic glomerulonephritis (Schaier et al., 2001). ATRA therapy further reduces lymphocyte proliferation and glomerulonephritis in MRL/lpr mice (Pérez de Lema et al., 2004). The protective role of retinoids has also been documented in mice with diabetic nephropathy (Han et al., 2004) and in models of antibody-mediated podocyte injury (Yao et al., 2013). ATRA restores the expression of podocyte differentiation markers, including nephrin, podocin, and synaptophysin, *in vivo* (Vaughan et al., 2005). However, systematic bibliometric analysis of retinoic acid research in the kidney domain is lacking.

**FIGURE 1:**
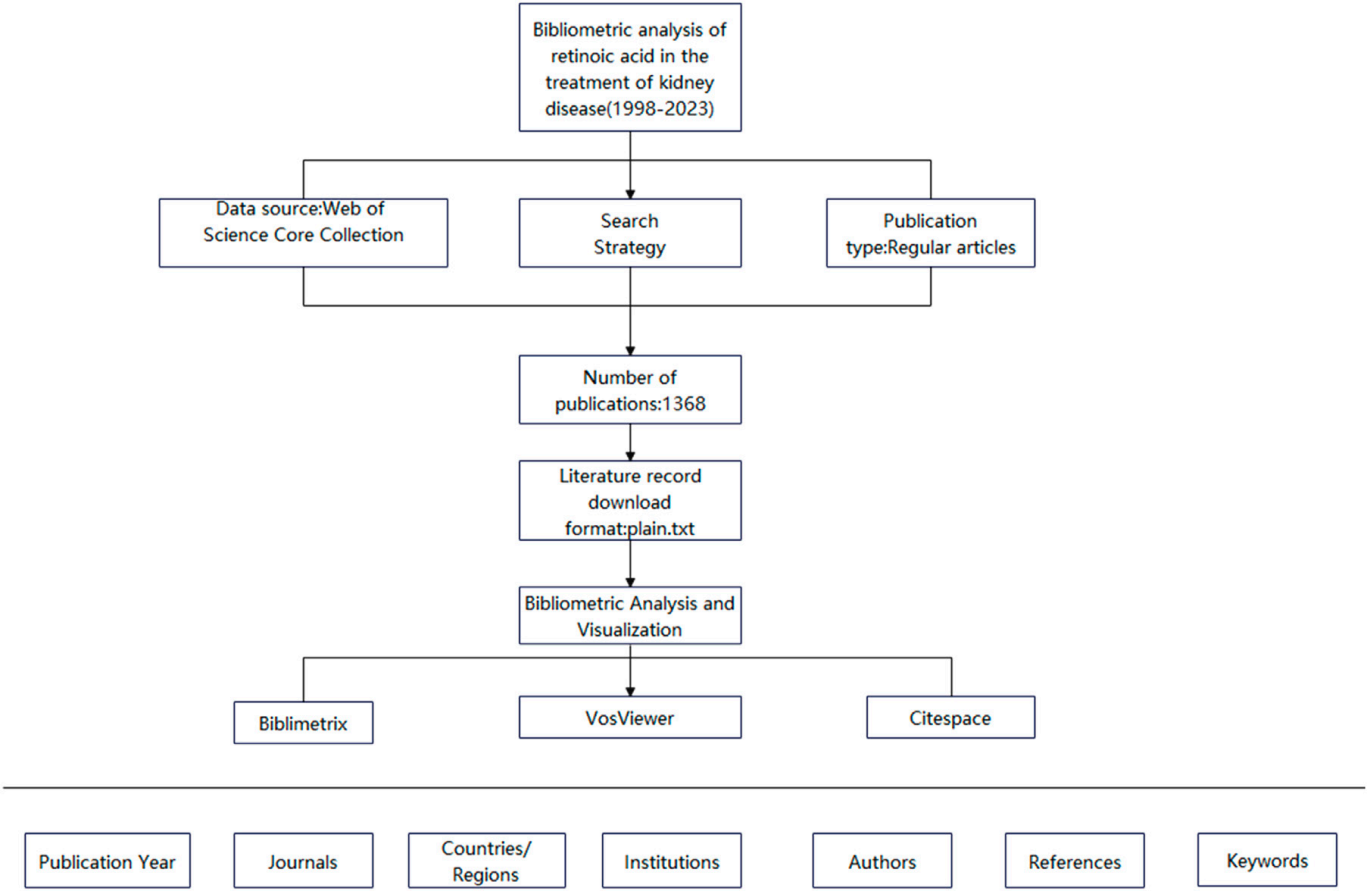
Flow chart.

Bibliometrics involves studying the distribution structure, quantitative relationships, and changing patterns of literature and bibliometric features via mathematical and statistical methods for the quantitative management and analysis of literature and information (Yao et al., 2013). It can be used to investigate science, technology, and certain structures, characteristics, and laws related to them. VOSviewer is a software tool based on an algorithm that clusters high-frequency text keywords. It can thus reflect research themes within a discipline and present the relationships between these themes in a manner that is easy to understand. CiteSpace is a visualization software used for literature analysis and constructing literature knowledge maps and coword maps to identify connections and patterns among publications accurately. Owing to the availability of large amounts of data in the information age, researchers have applied bibliometric methods combined with visualization analysis software to conduct bibliometric analyses across various fields. However, bibliometrics has not been used to investigate retinoic acid’s effectiveness in treating kidney diseases. Therefore, in this study, we used bibliometric methods, searched the Web of Science database, and used the VOSviewer and CiteSpace visualization software applications to synthesize and analyze the literature on retinoic acid. Our findings might serve as a reference for future research (Figure 1).

## 2 Methods

### 2.1 Literature inclusion

The inclusion criteria for the literature were as follows: published studies were collected through the Web of Science Core Collection on 24 March 2023, using the search terms TS = (retinoic acid or tretinoin) and TS = (kidney or renal) with the language set to English. Articles and reviews were included for analysis. After the data were standardized and verified, the studies, including full records and referenced citations, were exported in plain text format.

### 2.2 Data analysis

In this study, we used VOSviewer (version 1.6.18) to analyze countries and institutions, authors and coauthors, journals and cocited journals, and keyword co-occurrence. In the networks produced by VOSviewer, each node represents an entity. The size and color of a node indicate the quantity and category of these entities, respectively. The thickness of the lines between nodes reflects the degree of cocitation or collaboration between entities. We used CiteSpace (version 6.1. R6) to create dual-map overlays of journals for analysis. The “bibliometrix” R package (version 3.2.1) was used to construct a global distribution network in the field of retinoic acid treatment for kidney diseases and conduct thematic evolution analysis. Microsoft Office Excel 2019 was used for the quantitative analysis of publications.

## 3 Results

### 3.1 Publication quantity analysis

**FIGURE 2:**
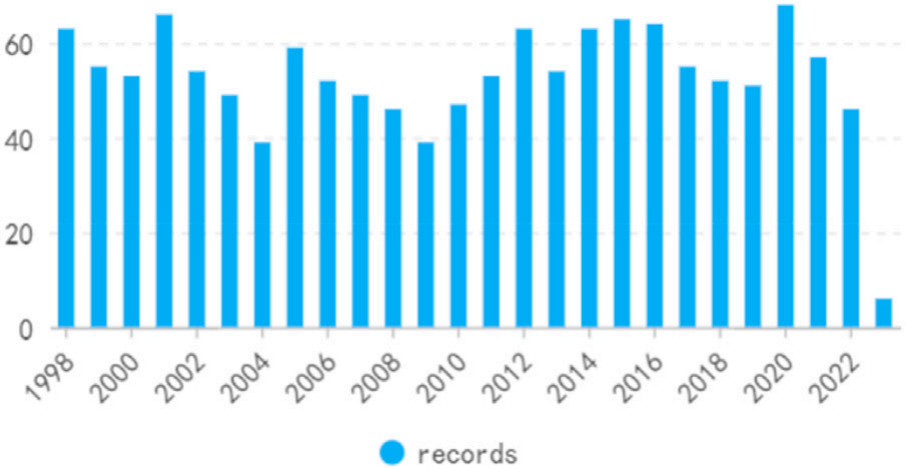
Publication volume.

**FIGURE 3:**
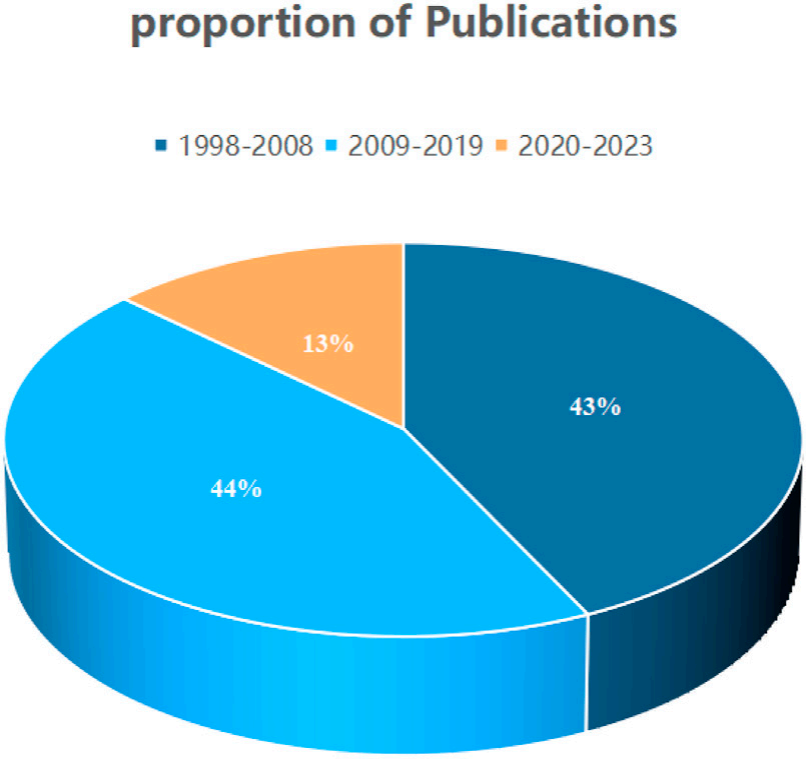
Publication volume percentage.

On the basis of our defined search criteria, from 1998 to 2023, 1,368 studies concerning the treatment of kidney diseases with retinoic acid, comprising 1,216″articles” and 152“reviews”, were identified (Figures 2, 3). The first article on this subject was published in the Web of Science in 1998. The publication volume revealed that 585 articles (43%) were published from 1998 to 2008, 606 articles (44%) were published from 2009 to 2019, and 177 articles (13%) were published from 2020 to 2023.

### 3.2 Country and institutional analysis

**FIGURE 4:**
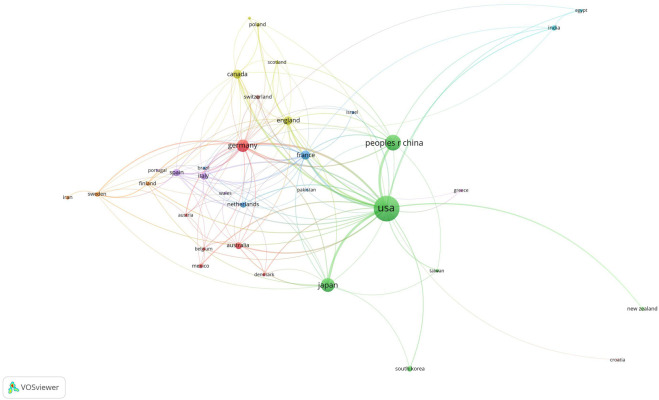
Visualization of countries associated with research on retinoic acid treatment for kidney diseases.

The publications on retinoic acid treatment for kidney diseases originated from 35 countries and 95 institutions. The top 10 countries were distributed across North America, Europe, and Asia, with the majority being in North America (n = 2) and Europe (n = 5) (Figure 4; Table 1). Among these countries, the United States published the greatest number of articles (n = 479), followed by China (n = 183), Japan (n = 144), and Germany (n = 114). We then filtered and visualized the 35 countries with publication counts of five or more and constructed a collaboration network based on the number and relationships of publications per country. Many international collaborations, such as those between China and the United Kingdom, France, and Canada, as well as active collaboration between the US, China, Japan, and Germany, have also been reported.

**TABLE 1:** Top ten ranked countries and institutions.

Rank	Country	Counts	Institution	Counts
1	USA	479	University of Notre Dame (USA)	31
2	China	183	Cornell University (USA)	24
3	Japan	144	Harvard University (USA)	24
4	Germany	114	Guangxi Medical University (China)	21
5	France	59	The University of Tokyo (Japan)	20
6	Canada	59	Sun Yat-sen University (China)	19
7	England	53	Baylor College of Medicine (USA)	16
8	Spain	38	Columbia University in the City of New York (USA)	15
9	Australia	34	University of California, Berkel (USA)	15
10	Italy	34	Karolinska Institute (Sweden)	14

**FIGURE 5:**
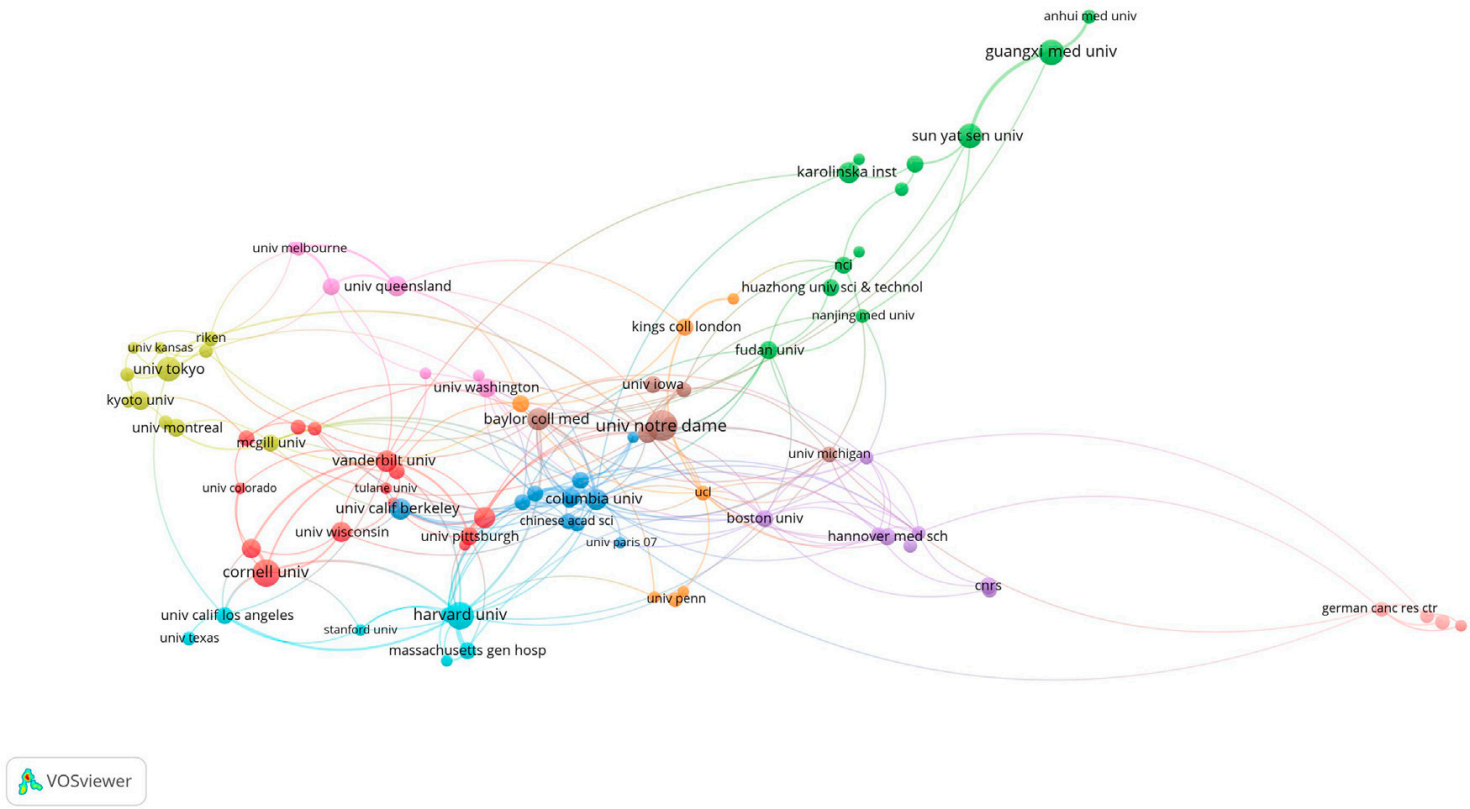
Visualization of research institutions.

The top 10 institutions were located in three countries, with the top three in the United States. The four institutions that published the most papers on this topic were the University of Notre Dame (n = 31), Cornell University (n = 24), Harvard University (n = 24), and Guangxi Medical University (n = 21). We subsequently visualized 96 institutions and built a collaboration network based on the number of papers published and the relationships between institutions (Figure 5). We found close cooperation between Cornell University, Columbia University, Baylor College of Medicine, and Harvard University, as well as between Sun Yat-sen University, Guangxi Medical University, and Baylor College of Medicine (Figure 5).

### 3.3 Journal and co-cited journals

**FIGURE 6:**
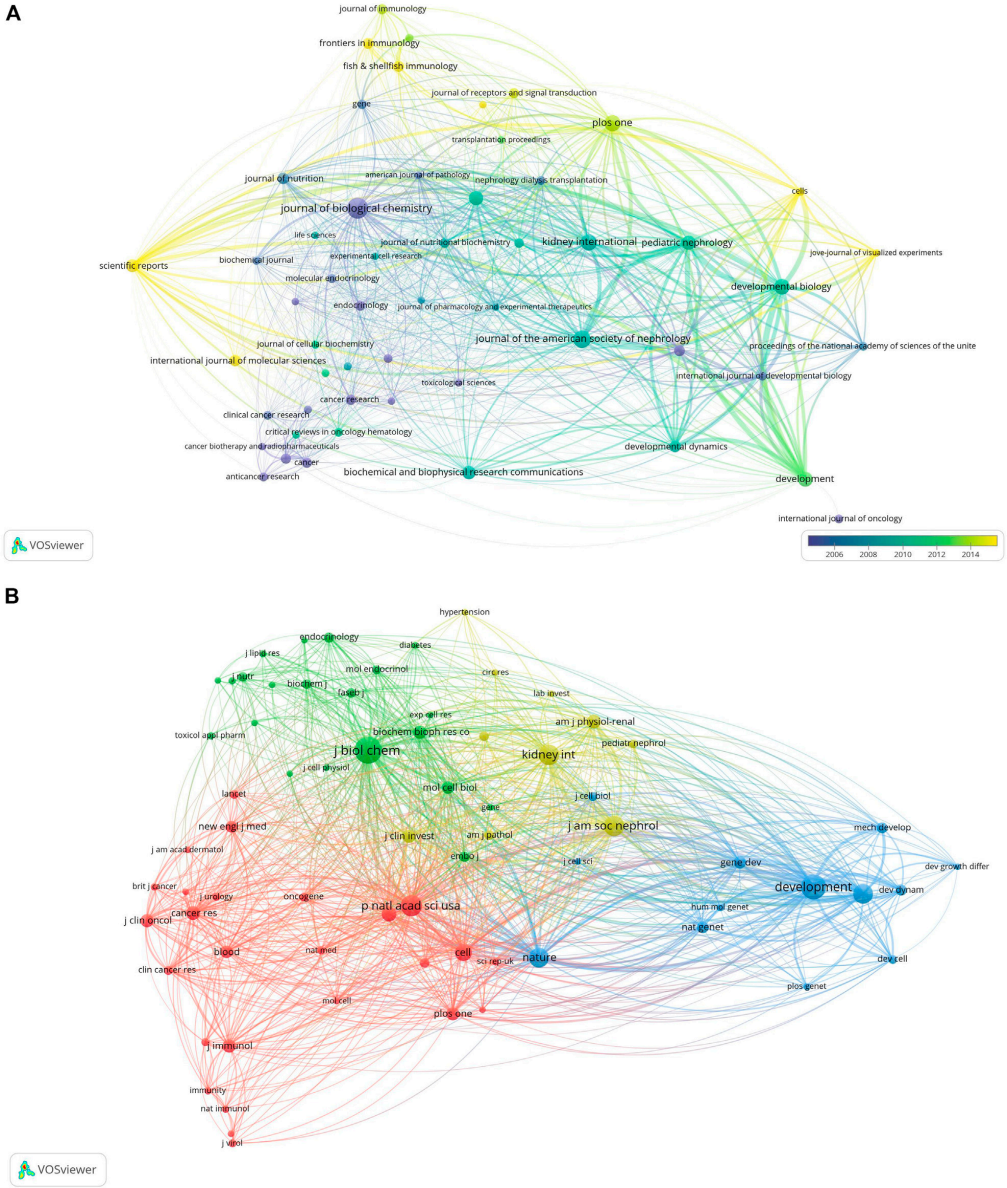
Visualization of journals related to research on retinoic acid treatment for kidney diseases **(A)** and cocited journals **(B)**.

Studies on retinoic acid treatment for kidney diseases were published across 54 different journals within the study period, with the JOURNAL OF BIOLOGICAL CHEMISTRY featuring the highest number of articles (n = 38, 2.7%), followed by the JOURNAL OF THE AMERICAN SOCIETY OF NEPHROLOGY (n = 27, 1.9%), PLoS One (n = 24, 1.7%), and KIDNEY INTERNATIONAL (n = 23, 1.6%). Among the top 15 journals in terms of publication volume, KIDNEY INTERNATIONAL has the highest impact factor (IF = 18.998), followed by the JOURNAL OF THE AMERICAN SOCIETY OF NEPHROLOGY (IF = 14.978). A journal network map was subsequently created. The JOURNAL OF BIOLOGICAL CHEMISTRY has active citation relationships with journals such as DEVELOPMENT, KIDNEY INTERNATIONAL, and PLoS One (Figure 6A, Table 2).

**TABLE 2:** The top 15 journals and cocited journals related to research on retinoic acid treatment for kidney diseases.

Rank	Journal	Count	If	Cocited journal	Cocitation	If
1	Journal of biological chemistry	38 (2.7%)	5.486	Journal of biological chemistry	2535	5.486
2	Journal of the American society of nephrology	27 (1.9%)	14.978	Development	2121	6.862
3	PLoS One	24 (1.7%)	3.752	Proceedings Of The National Academy Of Sciences Of The United States of America	1493	12.779
4	Kidney international	23 (1.6%)	18.998	Journal of the American society of nephrology	1460	14.978
5	Developmental biology	22 (1.6%)	3.148	Kidney international	1455	18.998
6	Development	19 (1.3%)	6.862	Developmental Biology	1321	3.148
7	American journal of physiology-renal physiology	18 (1.3%)	4.097	Nature	1296	69.504
8	Pediatric nephrology	18 (1.3%)	3.651	Cell	1000	66.850
9	biochemical and biophysical research communications	17 (1.2%)	3.651	Science	740	63.714
10	Scientific Reports	14 (1%)	4.996	American Journal of Physiology-renal Physiology	686	4.097
11	Journal of nutrition	12 (0.8%)	4.687	Cancer research	669	13.312
12	International journal of molecular sciences	12 (0.8%)	6.208	PLoS ONE	632	3.752
13	Developmental dynamics	12 (0.8%)	2.842	Biochemical And Biophysical Research Communications	616	3.322
14	Frontiers in Immunology	10 (0.7%)	8.786	Journal of immunology	614	5.426
15	Mechanisms of development	10 (0.7%)	1.81	Journal of clinical investigation	613	19.456

Among the top 15 cocited journals, eight were cited more than 1,000 times each; among them, the JOURNAL OF BIOLOGICAL CHEMISTRY received the highest number of citations (2,535), followed by DEVELOPMENT (cocitations = 2,121), Proceedings Of The National Academy Of Sciences Of The United States of America (cocitations = 1,493), the JOURNAL OF THE AMERICAN SOCIETY OF NEPHROLOGY (cocitations = 1,460), KIDNEY INTERNATIONAL (cocitations = 1,455), DEVELOPMENTAL BIOLOGY (cocitations = 1,321), Nature (cocitations = 1,296), and CELL (cocitations = 1,000). Moreover, Nature has the highest impact factor (IF = 69.054), followed by CELL (IF = 66.850). A cocitation network map was created for journals with at least 150 cocitations. The JOURNAL OF BIOLOGICAL CHEMISTRY showed positive cocitation relationships with journals such as DEVELOPMENT, the Proceedings of the National Academy of Sciences of the United States of America, and Nature (Figure 6B, Table 2).

**FIGURE 7:**
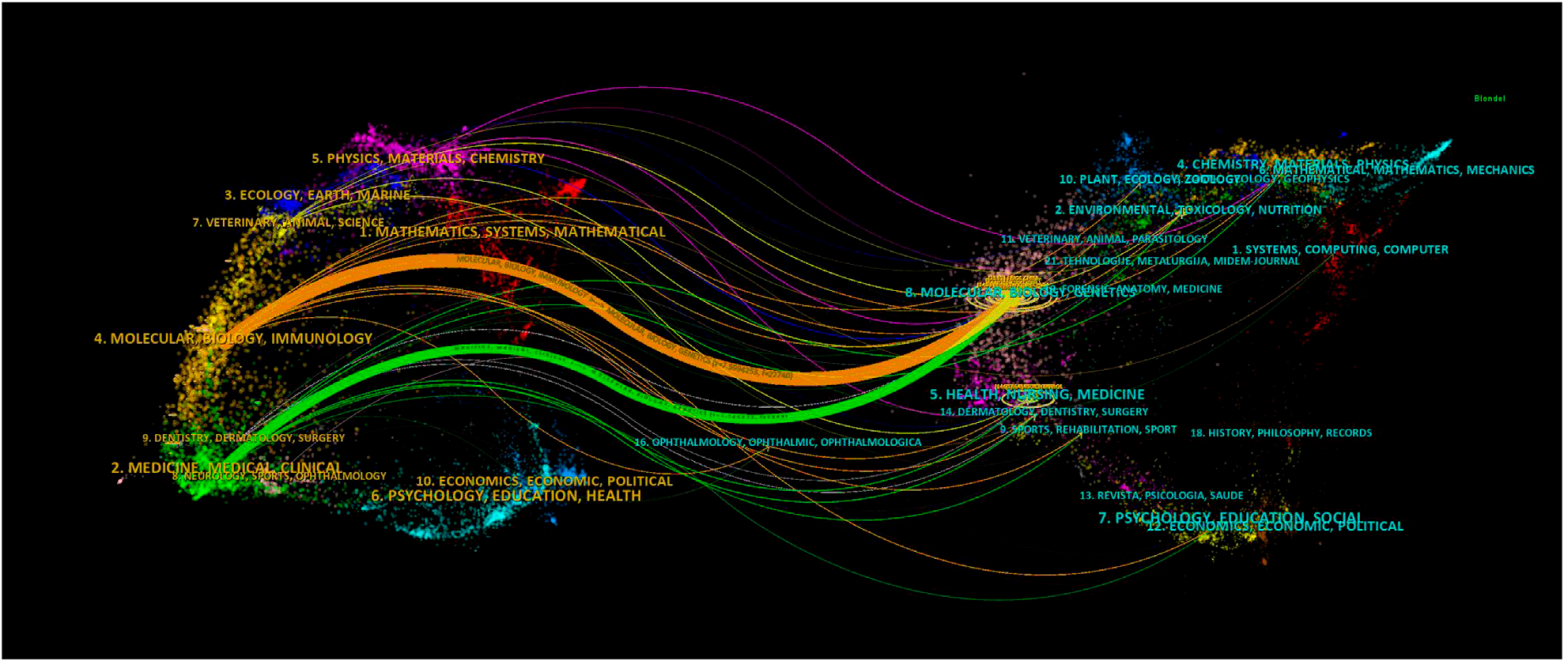
Dual-map overlay of journals related to research on retinoic acid treatment for kidney diseases.

Using dual-map overlays to illustrate the citation relationship between citing and cocited journals, with citing journal clusters on the left and cocited journal clusters on the right. The orange pathways represent the main citation pathways (Figure 7), indicating that studies published in molecular/biological/immunological journals are primarily cited by literature in molecular/biological/immunological journals.

### 3.4 Authors and co-cited authors

**FIGURE 8:**
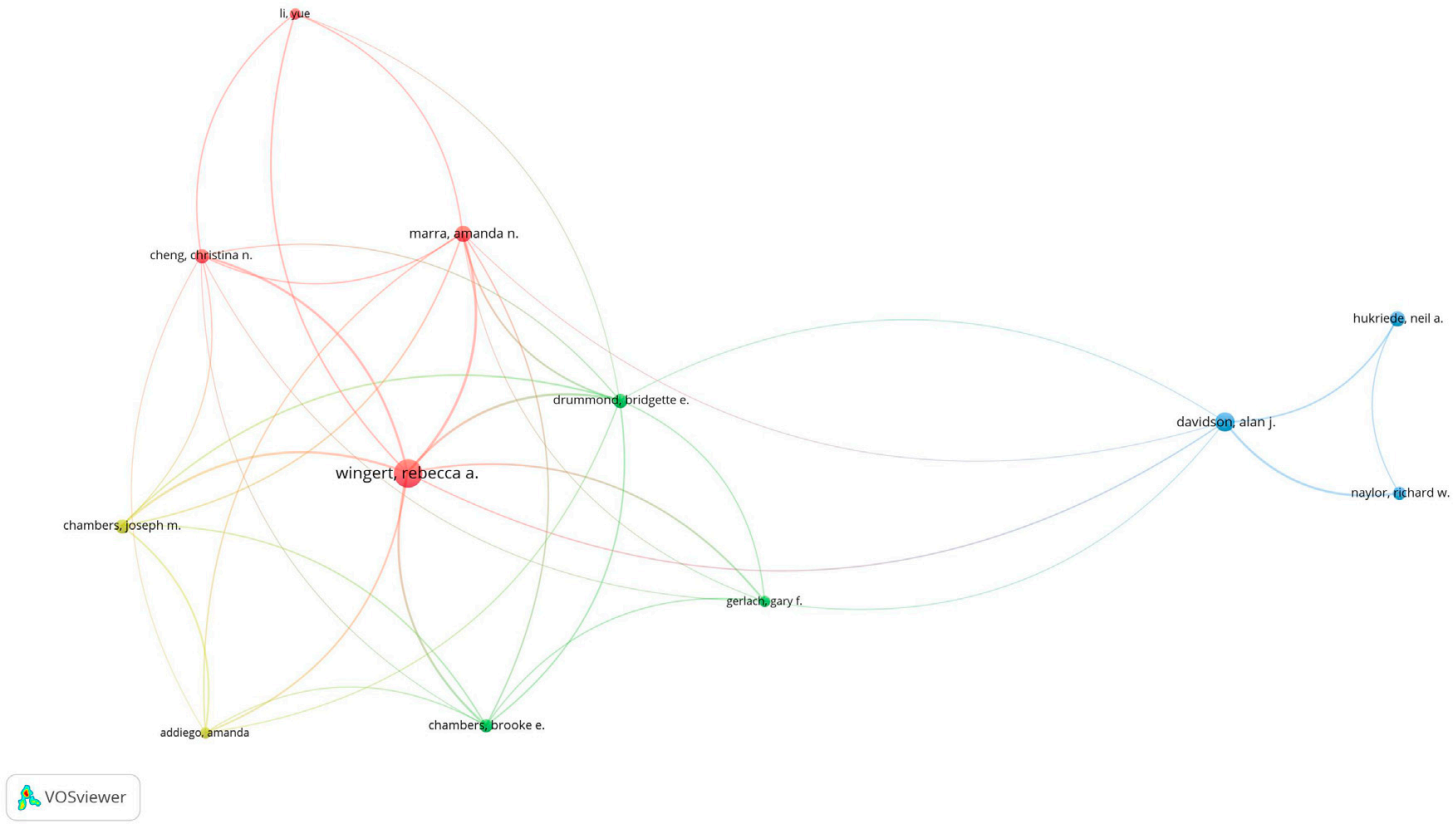
Visualization of authors who conducted research on retinoic acid treatment for kidney diseases.

In total, 69 authors contributed to the research on retinoic acid treatment for kidney diseases. Among the top 10 authors, four published more than 10 papers (Table 3). We constructed a collaboration network on the basis of the authors who published five or more papers (Figure 8). Wingert, Rebecca A, Zhou, Tian-biao, Qin, Yuan-han, Davidson, and Alan J were the authors with the largest nodes. Close collaboration was found among several authors. For example, Wingert, Rebecca A. closely collaborated with Marra, Amanda N, Marra, Amanda N, Cheng, Christina N, and Li Yue; Alan J. Davidson, Alan J collaborated with Naylor, Richard W, and others.

**TABLE 3:** Authors and cocited authors.

Rank	Authors	Count	Cocited authors	Citations
1	wingert, rebecca a	32	wingert, ra	179
2	zhou, tian-biao	20	motzer, rj	175
3	qin, yuan-han	18	niederreither, k	111
4	davidson, alan j	15	mendelsohn, c	109
5	tanaka, hiroshi	14	napoli, jl	94
6	imaizumi, tadaatsu	14	zhou, tb	89
7	nanus, dm	13	dressler, gr	82
8	yoshida, hidemi	12	bhat, pv	79
9	lei, feng-ying	12	wagner, j	77
10	gudas, lorraine j	12	chambon, p	75

**FIGURE 9:**
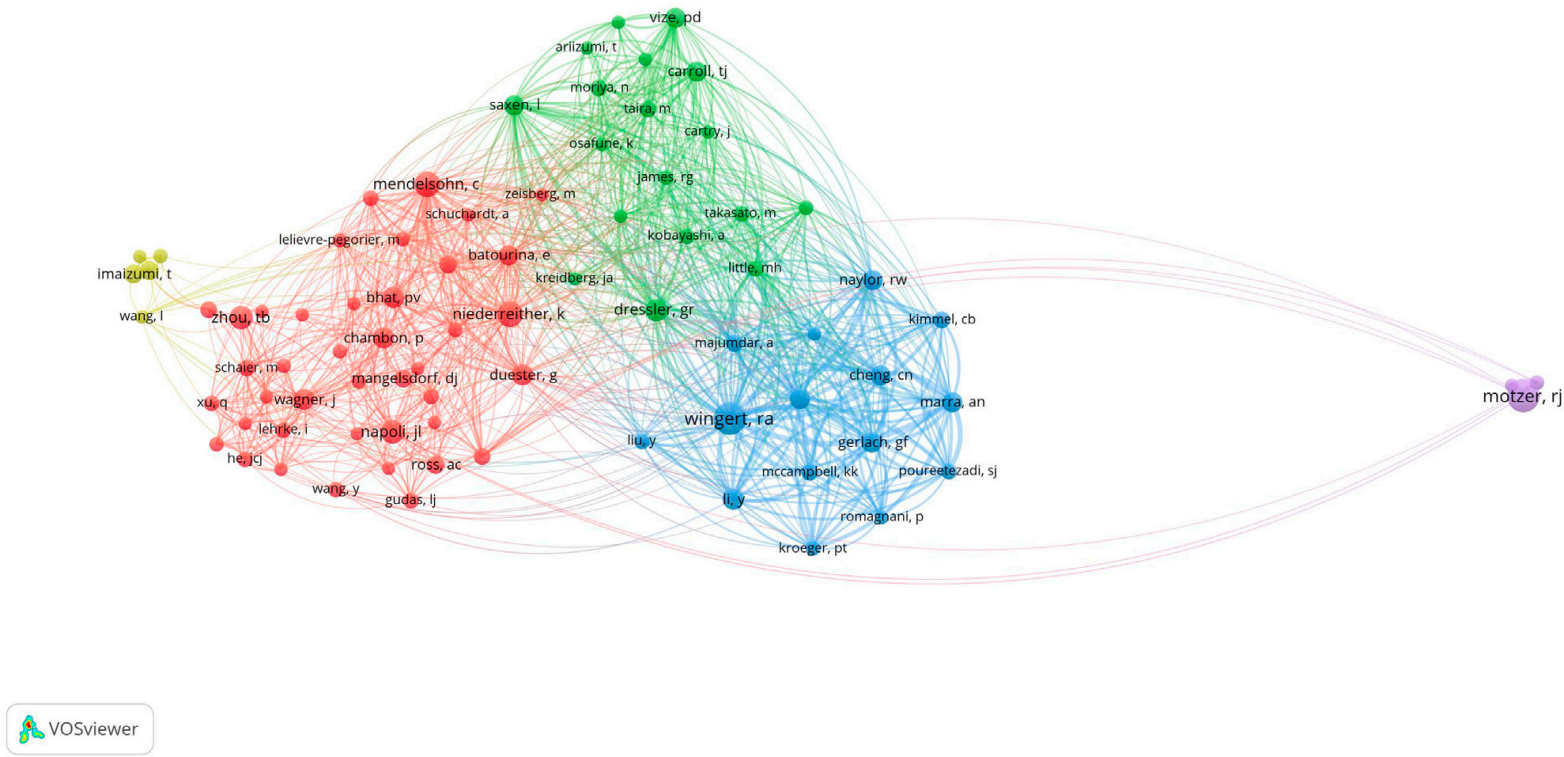
Visualization of cocited authors who conducted research on retinoic acid treatment for kidney diseases.

Among the cocited authors, four were cited more than 100 times (Table 3). Wingert, RA was the most cited, with 179 citations, followed by Motzer, RJ with 175 citations, and Niederreither, K with 111 citations. The authors with the minimum number of citations were selected for constructing a cocited author network map (Figure 9). Figure 9 depicts active collaborative relationships among different cocited authors, for example, between Motzer, RJ and Napoli, JL, and Mangelsdorf, DJ and Maden, M.

### 3.5 Cocited references

**FIGURE 10:**
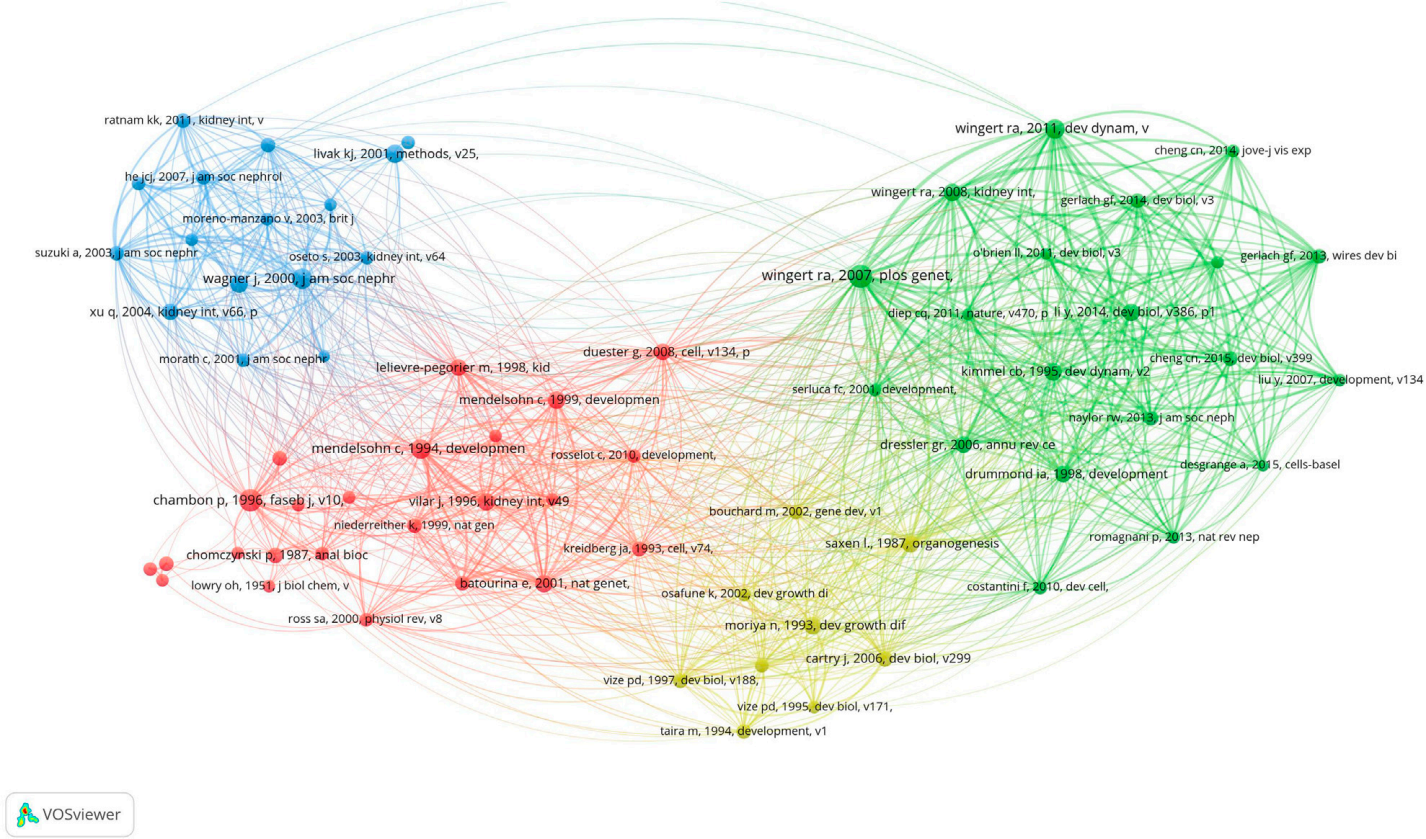
Visualization of cocited references related to research on retinoic acid treatment for kidney diseases.

We identified 69 cocited publications related to research on retinoic acid treatment for kidney diseases. Among the top 10 cocited publications (Table 4), all were cited at least 42 times, and one publication was cited more than 71 times. We selected publications that were cited 23 times or more to construct a cocitation network map (Figure 8). A positive cocitation relationship was found among several publications, including Wingert RA, 2007, PLOS Genet, v3, p1922; Wagner J, 2000, J Am Soc Nephrol, v11, p1479; Li Y, 2014, Dev Biol, v386, p111; and Wingert RA, 2011, Dev Dynam, v240, p2011 (Figure 10).

**TABLE 4:** The top 10 cocited references for the treatment of kidney disease with retinoic acid.

Rank	Cocited References	Citations
1	wingert ra, 2007, plos genet, v3, p1922, doi 10.1371/journal.pgen.0030189	71
2	chambon p, 1996, faseb j, v10, p940, doi 10.1096/fasebj.10.9.8801176	70
3	wagner j, 2000, j am soc nephrol, v11, p1479, doi 10.1681/asn.v1181479	58
4	wingert ra, 2011, dev dynam, v240, p2011, doi 10.1002/dvdy.22691	54
5	mendelsohn c, 1994, development, v120, p2749	51
6	livak kj, 2001, methods, v25, p402, doi 10.1006/meth.2001.1262	48
7	wingert ra, 2008, kidney int, v73, p1120, doi 10.1038/ki.2008.37	44
8	saxen l., 1987, organogenesis kidney	44
9	mendelsohn c, 1999, development, v126, p1139	43
10	li y, 2014, dev biol, v386, p111, doi 10.1016/j.ydbio.2013.11.021	42

### 3.6 Hotspots and frontiers

We quickly identified the research hotspots within a field by conducting keyword co-occurrence analysis. The top 20 high-frequency keywords related to the study of retinoic acid treatment for kidney diseases are listed in Table 5. Among these keywords, the terms “apoptosis” and “gene expression” appeared more than 20 times, representing the main research directions in the study of retinoic acid treatment for kidney diseases.

**TABLE 5:** The top 20 keywords related to retinoic acid treatment for kidney diseases.

Rank	Keywords	Count	Rank	Keywords	Count
1	retinoic acid	112	11	inflammation	22
2	kidney	86	12	Kidney development	19
3	vitamin a	45	13	*xenopus*	19
4	retinoids	37	14	chronic kidney disease	18
5	all-trans retinoic acid	34	15	retinol	18
6	pronephros	32	16	renal cell carcinoma	18
7	zebrafish	30	17	nephron	16
8	apoptosis	25	18	acute promyelocytic leukemia	15
9	gene expression	25	19	development	15
10	differentiation	23	20	retinoid	15

**FIGURE 11:**
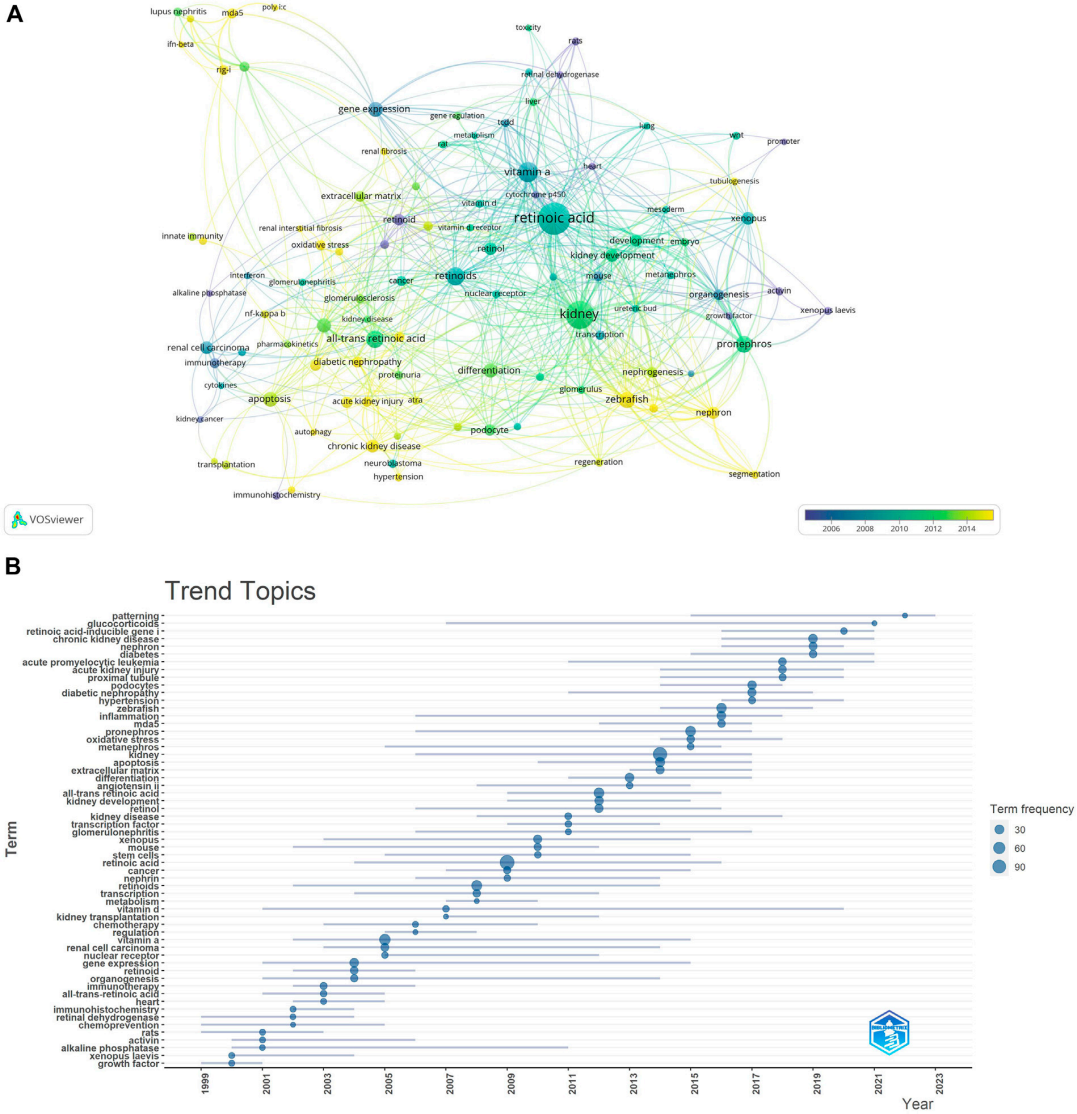
**(A)** Keyword clustering analysis. **(B)** Trend topic analysis.

We filtered keywords that appeared 10 times or more and conducted a clustering analysis via VOSviewer. We identified five clusters representing five research directions (Figure 11A). The keywords in the green cluster included acute kidney injury, chronic kidney disease, diabetic nephropathy, inflammation, diabetes, etc. The keywords in the red cluster included mesangial cells, mda5, podocytes, extracellular matrix, etc. The keywords in the blue cluster included differentiation, transcription, oxidative stress, etc. The keywords in the purple cluster included kidney development, mouse, vitamin A, etc. The keywords in the yellow cluster included *Xenopus*, kidney, and organogenesis. The results of the keyword trend theme analysis (Figure 11B) indicate that from 1999 to 2010, researchers focused primarily on immunotherapy, chemotherapy, and organ development, with key terms including immunotherapy, chemotherapy, kidney development, and organogenesis. Since 2011, scholars have explored retinoic acid’s pathogenesis and therapeutic potential in treating kidney diseases, with key terms such as kidney disease, diabetic nephropathy, chronic kidney disease, and all-trans retinoic acid. Additionally, in recent years, the frequency of keywords such as inflammation, cell apoptosis, and kidney has increased significantly, highlighting current research priorities for retinoic acid in treating kidney diseases. These findings highlight the timeline of research in this field, with the focus changing from the therapeutic effect to the underlying mechanisms.

## 4 Discussion

In this study, we utilized VOSviewer and CiteSpace software applications to analyze literature related to retinoic acid research in nephrology, reviewing the findings and advancements. We quantitatively analyzed the number of countries, authors, institutions, journals, and publications. From 1998 to 2023, a total of 1368 articles were published in the field of retinoic acid and kidney disease, with the highest number of publications—606 articles—between 2009 and 2019. We performed a statistical analysis of the number of papers published by various countries/regions and institutions, which identified key countries/regions and institutions with significant contributions to and impacts on retinoic acid treatments for kidney diseases, thereby elucidating their collaborative relationships. The United States, China, and Japan are the primary countries conducting research on retinoic acid in nephrology. Among the top 10 institutions, six are from the Netherlands, two are from the United States, and one is from Japan; among these, the University of Notre Dame led in publications. There is close collaboration between countries and institutions, and this exchange of information is instrumental in breaking down academic barriers and further advancing retinoic acid research in kidney disease.

Among the authors analyzed, Wingate, Rebecca A., Zhou, Tian Biao, Qin, Yuan Han, and Davidson, Alan J., published the most articles. Professor Rebecca A. Wingert primarily researched the impact of retinoic acid on kidney development in zebrafish. RA Wingert examined the anterior kidney of zebrafish and discovered that the transcription factors tbx2a/b are regulated downstream of the RA, segmenting the anterior kidney. The interaction between Notch signaling and tbx2a/b can regulate the formation of nephrons (Marra and Wingert, 2016). Two studies also suggested that retinoic acid influences fibroblast-like cells in zebrafish kidneys, thereby being essential for forming such cells. Zhou, Tian Biao, Qin, and Yuan Han (Konta et al., 2001; Wesselman et al., 2022) reported that retinoic acid can mitigate podocyte damage induced by doxorubicin and renal tubular epithelial cell damage due to hypoxia‒reoxygenation (Zhou et al., 2011). Other researchers have also reported that retinoic acid can treat interstitial fibrosis in rats, which is closely associated with a reduction in extracellular matrix accumulation. A study by Davidson, Alan J. indicated that retinoic acid is closely related to the development of kidney tissue (Kong et al., 2021). Overall, these studies have focused primarily on kidney development, the mechanisms of retinoic acid in treating kidney diseases, and related research areas.

Among the cocited authors, Wingert from RA (cited 179 times) is the most cited, followed by Motzer from RJ (cited 175 times) and Niederreither from K (cited 111 times). In 2011, progenitor cells in zebrafish kidney units underwent complex gene expression changes during development. Using a zebrafish model lacking retinoic acid, researchers discovered that retinoic acid regulates the formation of progenitor cells (Wingert and Davidson, 2011). In 2015, a study revealed that the dynamic expression of sim1a in renal progenitor cells is essential for directing the fates of PST and CS. The expression domain of sim1a in renal progenitor cells is responsive to changes in retinoic acid levels, suggesting that retinoic acid can directly or indirectly regulate sim1a during renal development (Cheng and Wingert, 2015). Emx1 was identified as a novel segmental regulator guiding tubular segment development. In 2018, retinoic acid was shown to regulate the expression of Emx1 (Morales et al., 2018) negatively. In 2019, Irx2a was identified as a key factor in the formation of branching and transport cells in zebrafish kidneys, with the retinoic acid signaling pathway modulating the expression of Irx2a (Marra et al., 2019). In summary, Wingert et al. established a theoretical and experimental foundation for studying kidney development using zebrafish as a model system, with a focus on the role of retinoic acid in this process.

Publications commonly cited by several researchers are regarded as foundational in a particular field. In this study, we chose the ten most frequently cited publications to identify the foundational research on treating kidney diseases with retinoic acid. The study most frequently cited was conducted by Wingert et al. (2007), who demonstrated that the cdx gene determines the localization of the anterior kidney through the axial positioning of retinoic acid activity (Wingert et al., 2007). In 2008, a review highlighted that retinoic acid signaling establishes proximal‒distal segment identity in zebrafish kidney precursors by regulating the expression of renal transcription factors and signaling pathway components that guide segment fate during mammalian kidney development. In collaboration with Mendelsohn C, researchers discovered that RARalpha and RARbeta2 are essential for initiating signals in mesenchymal cells that sustain the expression of C-ret in embryonic kidneys (Mendelsohn et al., 1999). Chambon et al. (1996) summarized the advancements in understanding the structure‒function relationship of retinoic acid, noting that retinoic acid and its receptors can bind to the cis-acting elements of many genes, thereby regulating gene expression and influencing cell differentiation, development, and homeostasis (Chambon, 1996). In 2000, Professor Wagner J reported that in established kidney injury models, all-trans retinoic acid can inhibit glomerular proliferation, damage to the glomerulus, and proteinuria (Wagner et al., 2000). These findings revealed the intricate interplay between retinoic acid and the transcription factor methylcobalamin in shaping the fate of proximal and distal tubular segments and multi-branched cells during the development of zebrafish kidney precursors (Li et al., 2014). In conclusion, the cited literature focuses primarily on the role of retinoic acid in kidney growth and development, establishing a foundation for research into retinoic acid and kidney diseases.

Keyword clustering and trend analysis can rapidly identify the distribution and evolution of research interest in the field of using retinoic acid to treat kidney diseases. After removing keywords such as retinoic acid, all trans retinoic acid, vitamin A, and kidney, the remaining keywords mostly pertain to zebrafish, foregut kidney, inflammation, cell apoptosis, and organ development. Retinoic acid significantly influences various biological processes, including cell proliferation, differentiation, and morphogenesis (Konta et al., 2001). However, research on the antiapoptotic effects of retinoic acid is limited. Researchers, including Moreno Manzano V (Moreno-Manzano et al., 1999), have reported that the antiapoptotic properties of all trans retinoic acid are partly due to the dual suppression of the JNK- and AP-1-mediated cell death pathways. Since zebrafish are nonamniotic vertebrates, their development can be observed from the single-cell stage (Hawkins and Wingert, 2023); moreover, they have a high reproductive capacity. These characteristics facilitate genetic screening and high-throughput analysis of the effects of various compounds. We previously discussed the role of retinoic acid in kidney development in sections on authors, cocited authors, and cocited publications, and will not repeat that information here. Renal inflammatory diseases are characterized by monocyte infiltration, which can cause significant damage. All trans retinoic acid, known for its potent ability to suppress monocyte adhesion and chemotaxis, is used to treat renal inflammatory diseases.

## 5 Limitations

This paper employs bibliometric methods to systematically analyze studies on the treatment of kidney diseases with retinoic acid, offering a theoretical foundation for researchers interested in this field. However, this study has several limitations. First, the data for this study are exclusively from the WOSCC database. Although the literature in the WOSCC database is comprehensive and reliable, it does not include all important studies, which were consequently excluded from our analysis. Second, this study included only English articles and reviews, omitting non-English publications and conference papers, which may introduce bias to the results. Third, the bibliometric analysis in this paper focuses on quantitative literature analysis rather than on evaluating and analyzing research quality. Fourth, the nature of the CiteSpace and VOSviewer tools may lead to inaccurate keyword extraction and incomplete article content analysis. Despite these limitations, the overall trends remain within an acceptable range.

## 6 Conclusion

In this study, we retrieved 1368 original articles concerning retinoic acid research in nephrology from 1998 to 2023, which were downloaded from the WOSCC database. Using VOSviewer and Citespace for data analysis, we obtained a thorough and unbiased understanding of the current state, trends, and cutting-edge developments in the field. Our findings reveal that the United States and China are the most prominent contributors to this field, with KIDNEY INTERNATIONAL and the JOURNAL OF THE AMERICAN SOCIETY OF NEPHROLOGY being the most prestigious journals for retinoic acid research in nephrology. Wen Ge Te, Zhou Tianbiao, Yuan Hanqin, and others stand out as the most influential scholars in this domain. The primary focus of researchers is to clarify the mechanisms by which retinoic acid affects kidney diseases. This research may provide future researchers with valuable information and accelerate the advancement of the field. In the future, more data need to be collected to examine the underlying mechanism of the therapeutic effect on kidney disorders. Moreover, clinical trials are needed to fully appreciate its potential.
